# Estimating the incidence of colorectal cancer in Sub–Saharan Africa: A systematic analysis

**DOI:** 10.7189/jogh.02.020204

**Published:** 2012-12

**Authors:** Alice Graham, Davies Adeloye, Liz Grant, Evropi Theodoratou, Harry Campbell

**Affiliations:** Centre for Population Health Sciences and Global Health Academy, The University of Edinburgh Medical School, Edinburgh, Scotland, UK

## Abstract

**Background:**

Nearly two–thirds of annual mortality worldwide is attributable to non–communicable diseases (NCDs), with 70% estimated to occur in low– and middle–income countries (LMIC). Colorectal cancer (CRC) accounts for over 600 000 deaths annually, but data concerning cancer rates in LMIC is very poor. This study analyses the data available to produce an estimate of the incidence of colorectal cancer in Sub–Saharan Africa (SSA).

**Methods:**

Data for this analysis came from two main sources: a systematic search of Medline, EMBASE and Global Health which found 15 published data sets, and an additional 42 unpublished data sets which were sourced from the IARC and individual cancer registries. Data for case rates by age and sex, as well as population denominators were extracted and analysed to produce an estimate of incidence.

**Results:**

The crude incidence of CRC in SSA for both sexes was found to be 4.04 per 100 000 population (4.38 for men and 3.69 for women). Incidence increased with age with the highest rates in Southern Africa, particularly in South Africa. The rates of CRC in SSA were much lower than those reported for high–income countries.

**Conclusion:**

Few health services in SSA are equipped to provide timely diagnosis and treatment of cancer in SSA. In addition, data collection systems are weak, meaning that the available statistics may underestimate the burden of disease. In order to improve health care services it is vital that accurate measurements of disease burden are available to policy makers.

In 2008 cancer was the leading cause of mortality worldwide, responsible for the deaths of an estimated 7.6 million people [1]. Colorectal cancer (CRC) accounted for over 600 000 of those deaths, with 70% occurring in low– and middle–income countries [1,2]. With the emergence of non–communicable diseases (NCD) in countries where traditionally the biggest problem were infections, it is estimated that, by 2030, cancer will become the cause of over 13 million deaths a year [3]. While rates of infectious diseases typically decrease with the economic growth of a country, rates of NCD do not appear to decrease until high levels of education and literacy are reached [2]. The increasing prevalence of NCD is also having serious economic impact on health systems around the world, with the cost of long–term treatment for chronic conditions unsustainable in many health systems [3]. NCDs are heavily involved in the vicious cycle of health and poverty, where poor health results in loss of income, which in turn results in inability to pay for health care or maintain a healthy lifestyle [4]. Many NCDs result in chronic conditions requiring long–term medical expenditures and ongoing loss of income owing to ill health [5].

Low– and middle–income countries (LMICs) carry the majority of the burden of NCDs both in terms of incidence and mortality [6]. The rates of cancer are dramatically increasing partly because of the ageing population, and partly due to the rapid ‘globalisation’ and the adoption of the associated risk factors within these populations [7]. These risk factors include physical inactivity, smoking and alcohol consumption and poor nutrition [8]. A recent study found that over half of people aged 50 or over in SSA possessed at least 2 of the risk factors associated with NCDs [7].

Cancer registries cover less than 25% of the world’s population. It is estimated that this proportion would reduce to 11% if only data of good quality is included [8]. The WHO collects data on cancer deaths from cancer registries around the world (**Box 1**) and produces estimates of the global and regional burden of cancer [14]. The International Agency for Research on Cancer (IARC) also publishes sets of estimates of global incidence and mortality through the GLOBOCAN project, the most recent from 2008 [15]. This data indicates that colorectal cancer (CRC) is the 5th most common cancer in SSA [15].

Box 1 **Non–communicable diseases and the global health agenda**

In recent decades the focus of international health organisations for the developing world has been communicable diseases. As a result huge sums of money and political attention have been dedicated specifically to HIV/AIDS, malaria and tuberculosis. However, NCD are now a significant barrier to reaching some of the Millennium Development Goals (MDGs), which focus primarily on reducing infectious diseases [5].

In 2000, the resolution “Prevention and Control on Non–Communicable Diseases” was adopted by WHO member states at the World Health Assembly [9]. This resolution had three main aims:

1. Examine the major risk factors associated with NCDs

2. Introduce health promotion programs aiming to minimise the risk factors

3. Improve access to health care

Since this initial commitment to reducing the burden of disease caused by NCDs the World Health Assembly has also endorsed the Global Strategy on Diet and Physical Activity and Health [10,11], and the Framework Convention for Tobacco Control [12].

The UN high–level meeting on non–communicable diseases in 2011 recognised four major conditions that had previously been neglected by the international health community; cardiovascular disease, chronic respiratory diseases, diabetes and cancer [4,13].

Although the reliability of the information provided in cancer registries, especially in SSA, is open to question, the registries remain one of the very few sources of data on cancer and therefore are the closest we can get to an estimate of the burden [8]. The size of the population denominator and the accuracy of the data can vary greatly between these sources, with only 23 of the 47 SSA countries having a formal registration system for cancer [16]. Many of these registries only cover small regions, often cities, within a country. Typically, the few rural registries indicate a much lower incidence of cancer than urban registries. Given that the majority of accessible data comes from urban registries, where only 40% of the population live and with higher risk factors, the estimates may be exaggerated [17] or gravely under–representative of rural areas.

Although the number of cases of CRC in SSA is thought to be very low in comparison to those diagnosed in the Western world (**Box 2**), it constitutes a significant proportion of the cancers in this region [24]. The aims of this study were to contribute to improving the evidence on the burden or NCDs in LMICs, by reviewing the evidence from the published literature (found through systematic review) and unpublished data on cancer registries to assess the burden of CRC in SSA. We also aimed to explore the quality and availability of data and to make suggestions for research and public health policy priorities to improve control of CRC in SSA.

Box 2 **Colorectal cancer and public health**

Commonly CRC develops from adenomatous, colonic polyps with 65% in the rectosigmoid and 15% in the ascending colon or caecum [18]. Spread of the cancer is typically through the bowel wall and, in cases of rectal carcinoma, may invade the abdominal walls or pelvic viscera. Lymphatic invasion through the systemic or portal circulation is common with the liver and lungs as secondary sites [19]. The progression from adenoma to carcinoma is a well–documented process involving mutations in a number of genes such as APC (adenomatous polyposis coli), DCC, k–ras and p53 [18,20].

Approximately 80% of CRC is caused by environmental factors such as physical inactivity, smoking, alcohol and poor diet [21]. Nutrition is thought to be a major contributing factor to the differences in incidence of CRC between developing and developed countries. Western diets, typically high in fibre, increase faecal bulk, reducing transit time and resulting in a higher risk of developing malignancy [21]. The other 10% of CRC is caused by genetic mutations in two major pathways resulting in familial adenomatous polyposis (FAP) or hereditary non–polyposis colon cancer (HNPCC). Individuals with these diseases have a lifetime risk of CRC of up to 80% [21].

Screening for CRC is known to not only help detect the cancer in its early stages, and therefore improve chances of a curative treatment, but also to detect pre–cancerous lesions which, if removed successfully, can avoid more expensive and radical treatments [22]. Colorectal adenomas/polyps are extremely common in the Western world, occurring in approximately 20% of people over the age of 60 [21]. In the UK a national screening programmes for CRC have been launched, targeting those in the 50–74 age group. It is estimated that these interventions have resulted in a 16% reduction of mortality caused by CRC [23].

## METHODS

**Figure 1:**
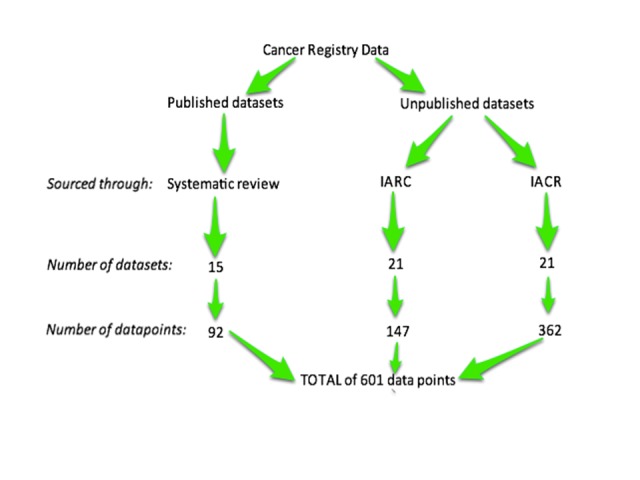
Sources of data.

The data in this study came from cancer registries, and were identified through two main sources – a systematic review of the published literature and an analysis of the unpublished cancer registry data, as shown in **Figure 1**.

### Search strategy for systematic analysis and data extraction

A systematic analysis of published literature on public domain was carried out using the databases Medline, Embase and Global Health. The search used both Medical Subject Headings (MeSH) and keywords as well as the individual countries in Sub–Saharan Africa. Search terms for Medline are outlined in **Table 1** and were modified where necessary for the other databases. Final searches were completed on 13 January 2012. All references found in the initial searches were exported to Refworks.

**Table 1:** Search terms

1	prevalence/ or prevalen*.tw
2	mortality/ or mortal*.tw
3	global burden of disease/ or (disease adj3 burden*).tw
4	incidence/ or inciden*.tw
5	1 or 2 or 3 or 4
6	colorectal adenoma/ or CRC/ or colon cancer/ or rectum cancer/ or colorectal carcinoma/ or colorectal disease/
7	(bowel* or large intestine* or large bowel* or gut* or colorect* or colo* or rect*) adj3 maligna* or carcinoma* or neoplas* or cancer* or tumo* or polyp*).tw
8	6 or 7
9	SSA/ or (Africa* adj3 Sub–Sahara*).tw or (Africa* adj3 south* adj3 Sahara*).tw
10	exp Africa or central/ or exp Cameroon/ or exp Central African Republic/ or exp Chad/ or exp Congo/ or exp “Democratic Republic of the Congo”/ or exp Equatorial Guinea/ or exp Gabon/ or exp Africa, Eastern/ or exp Burundi/ or exp Djibouti/ or exp Eritrea/ or exp Ethiopia/ or exp Kenya/ or exp Rwanda/ or exp Somalia/ or exp Sudan/ or exp Tanzania/ or exp Uganda/ or exp Africa, Southern/ or exp Angola/ or exp Botswana/ or exp Lesotho/ or exp Malawi/ or exp Mozambique/ or exp Namibia/ or exp South Africa/ or exp Swaziland/ or exp Zambia/ or exp Zimbabwe/ or exp Africa, Western/ or exp Benin/ or exp Burkina Faso/ or exp Cape Verde/ or exp Cote D'Ivoire/ or exp Gambia/ or exp Ghana/ or exp Guinea/ or exp Guinea-Bissau/ or exp Liberia/ or exp Mali/ or exp Mauritania/ or exp Niger/ or exp Nigeria/ or exp Senegal/ or exp Sierra Leone/ or exp Togo/ or exp Comoros/ or exp Madagascar/ or exp Mauritius/ or exp Seychelles
11	9 or 10
12	5 and 8 and 11
13	Limit 13 to yr = 1980-current

The selection criteria used in screening relevant studies were: original studies, limited to post–1980, conducted in Sub–Saharan Africa as defined by the World Bank [25], involving any age–group or sex, with no restrictions on the language of publication. The retained studies were further evaluated for quality of design and methods; studies with clear case definition of CRC, a population denominator of more than 10 000, and numerical measure of disease frequency were included.

Given that the systematic review produced a limited number of relevant papers, we decided to search for other potential sources of information on CRC in SSA. It was predicted that many of the cancer registries in SSA might hold unpublished or more recent data that could be obtained through methods other than systematic review. The IARC Web site provided access to an unpublished document presenting the data from cancer registries across SSA. Further review of this paper showed that some of the data had already been used in published articles found through the systematic search. However, there were an additional 21 data sets that met the criteria for this review. Permission to use this further data for analysis in this review was sought from the IARC through the World Health Organization (WHO). This was granted and is presented in Online Supplementary Document(Online Supplementary Document), where a sample of the data extracted from this IARC paper is also shown.

Contact details for the directors of cancer registries in SSA were provided on the IACR Web site [16]; they were contacted to investigate whether they had any unpublished data that could be included with permission. Of the 23 directors contacted, 11 replied. Two replies directed attention to the IARC document, mentioned above, that had the most recent data for their registries. Seven of the replies, although positive and encouraging, provided no additional data. The reply from Botswana contained data for CRC from 1998 to 2011. The reply from the South African national cancer registry provided details of their Web site which had extensive information on cases of CRC between 2000 and 2004 and population data for 2000–2002. Population estimates for 2003 and 2004 were obtained through the South African government statistics Web site [26]. This second additional source provided the current review with a further 21 datasets. Examples of the data received directly from cancer registries are presented in appendices 6 and 7. Owing to the extensive data available for South Africa a separate analysis was carried out investigating incidence trends over a 5–year period for different racial groups [27-31]. All the unpublished cancer registry data, both from the IARC and the IACR, was screened using the same selection criteria employed in the systematic literature search to ensure that the data was of comparable quality.

**Figure 2:**
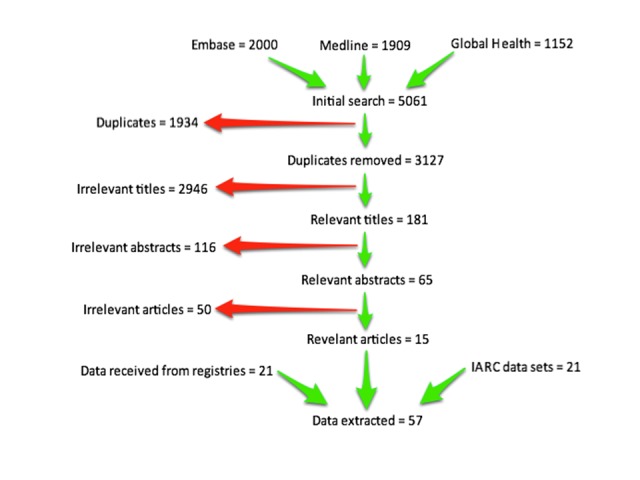
Search strategy.

The initial search of Medline, Embase and Global Health databases returned 3127 articles, after the removal of duplicates, as shown in **Figure 2**. Sixty–five articles were sourced for the full–text version, and of these, 15 were selected for inclusion in the review. The 50 articles considered irrelevant did not comply with the exclusion/inclusion criteria set for this review; however, some were retained for extra information, mainly in the discussion. The 15 relevant articles [10,20,32-42] were then supplemented by the 21 data sets secured with permission from the IARC [43,44] and the further 21 data sets which were received from the directors of the cancer registries through the IACR [16]. Six articles, which were not suitable for contribution to the estimates of incidence but were sourced for full–text copies, were retained for information on the biological characteristics of CRC diagnosed in SSA. Data from the articles was extracted and complied into spreadsheets in Microsoft Excel. Cases of CRC were separated into age groups and by sex where appropriate. Incidence estimates were either extracted from the included data sets or calculated using the data reported. All estimates were converted to incidence per 100 000 of population (in that sex and age group) per year to allow for direct comparison between results. Data from 6 additional articles, not used for calculating incidence, were extracted for more detailed information on the biological characteristics of CRC.

### Case definition used in retained studies

**Figure 3:**
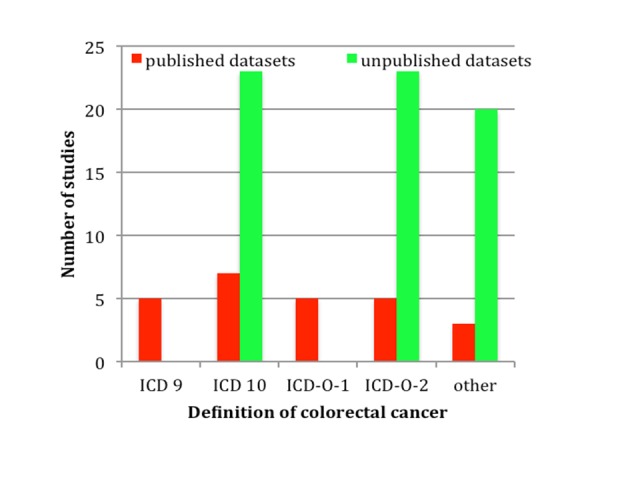
Definition of colorectal cancer in data sets. ICD – International Classification of Diseases (World Health Organisation).

The different definitions of CRC cancer used in the data sets are presented in **Figure 3**. The majority defined it based on the current International Classification of Diseases (ICD–10), as devised by the World Health Organisation [45]. Five data sets, the ones typically undertaken in the 1980s and early 1990s, defined CRC based on the earlier ICD–9. The remaining data sets (labelled ‘other’ in **Figure 3**) took retrospective data from cancer registries and did not give a specific definition of CRC. However, 20 of these came from the South Africa Cancer Registry and it would be reasonable to assume they may have used ICD–10. The International Classification of Diseases for Oncology (ICD–O) was also used in the diagnosis of CRC for nearly 60% of the data sets, with five using the 1st edition (ICD–O–1) and 28 using the 2nd edition (ICD–O–2).

### Data analysis

There were a total of 57 data sets available from the published articles (identified by the systematic review) and the unpublished data from the cancer registries. This provided a total of 601 datapoints for the calculation of incidence. In this pooled series, 28 data sets contained statistics for the population of the area covered by the registry separated by age and sex. They also gave the raw data on the number of cases of CRC diagnosed over in given time period, also separated by age and sex. This allowed for a simple calculation of the incidence for each sex and age group. Five of the data sets did not contain information on the population denominator for each age group. In these cases the total population was either cited or could be calculated by working back from the reported incidence and cases of CRC. This number was then separated into age groups using total population data for the relevant year and country and adjusted to the size of the study population.

In 3 data sets which neither presented the population denominator, nor showed the number of cases of CRC by age group, the average age of the population (separated by sex) was calculated using UN ESA data for the relevant year and country [46]. This was then combined with the overall reported incidence of CRC by sex, calculated using the above method. A few data sets presented the number of cases separately for colon and rectal cancer. However, data for the two were combined to allow for easier comparison with other data sets. Tables were compiled using Microsoft Excel and contained all the information extracted from the data sets (see Online Supplementary Document(Online Supplementary Document) for further details).

The average age was calculated as the mean age of each age range as reported in the data sets. This resulted in a total of 601 data points of incidence against age, separated by sex. Graphs containing the 601 data points of incidence by age for male, female and both sexes were used to separate incidence estimates into age groups. The age ranges were based on the groups used by the data sets from the IARC. From this, the minimum, maximum, lower quartile, upper quartile and median could be calculated for each age group. This data was used to create box–and–whiskers plots of the collected information. The median values of incidence by age group were then combined with the UN ESA statistics for the total population of SSA to calculate the number of new cases in a year. Examples of this are presented in Online Supplementary Document(Online Supplementary Document) along with the all–age data that were calculated by each data set.

Moreover, twenty data sets from the South African cancer registry were used to conduct a separate sub–analysis of time trends in incidence of CRC in South Africa from 2000 to 2004. These papers also provided valuable data on the relationship between ethnic group and incidence of CRC. Furthermore, data from 6 articles excluded from the systematic review nevertheless had useful background information on three aspects of CRC: presenting symptoms, anatomical site of tumour and Duke’s Stage of cancer at diagnosis. These papers provided percentage distributions, from which the averages could be calculated.

To ensure that the data was extracted accurately from both the published and unpublished data sets, one author (AG) performed a second data extraction on a random selection of 8 data sets. The number of cases of CRC as well as the underlying population denominator was extracted again. This represented 112 of the total 601 data points, and found that 100% of the data was the same. Therefore it was concluded that the accuracy of the data extraction was high.

## RESULTS

**Figure 4:**
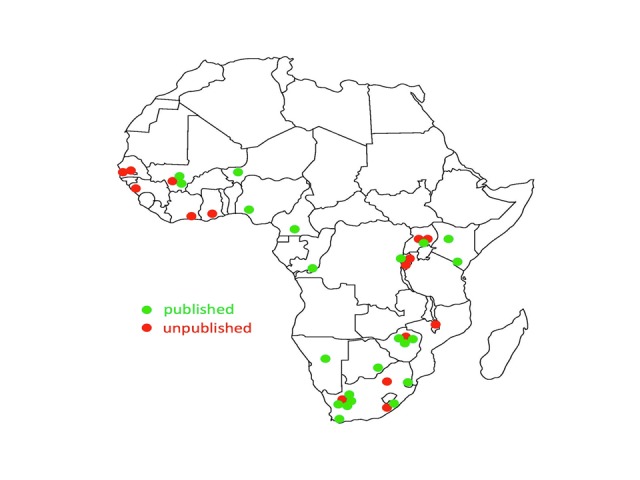
Geographical distribution of data sets. Additional 20 unpublished data sets came from South African Cancer Registry (not shown).

**Figure 5:**
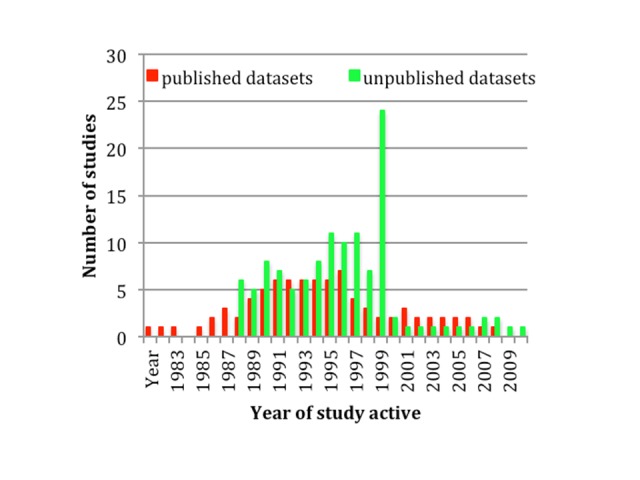
Active data sets active in study years.

The data sets showed a wide geographical distribution in SSA, as shown in **Figure 4**. Approximately 5% of data sets came from Central Africa, and 15%, 55% and 25% from West, Southern and East Africa respectively. However, clusters of data sets came from larger cities or countries known to conduct high levels of research. All data sets reported cancers from all age groups. The average size of the population denominator covered by the cancer registries was approximately 2 million. Ninety percent of the data sets reported data from population–based cancer registries, the remaining six were hospital–based. Some of the national cancer registries combined data from regional hospitals and smaller registries across the country. **Figure 5** shows the distribution of active data sets over time. The majority were conducted in the late 1990s to early 2000s with very little data available for the 1980s and a limited number from the last decade. As a result, any estimates produced by this review should be regarded as referring to 2000.

### Incidence of CRC in Sub–Saharan Africa

**Figure 6:**
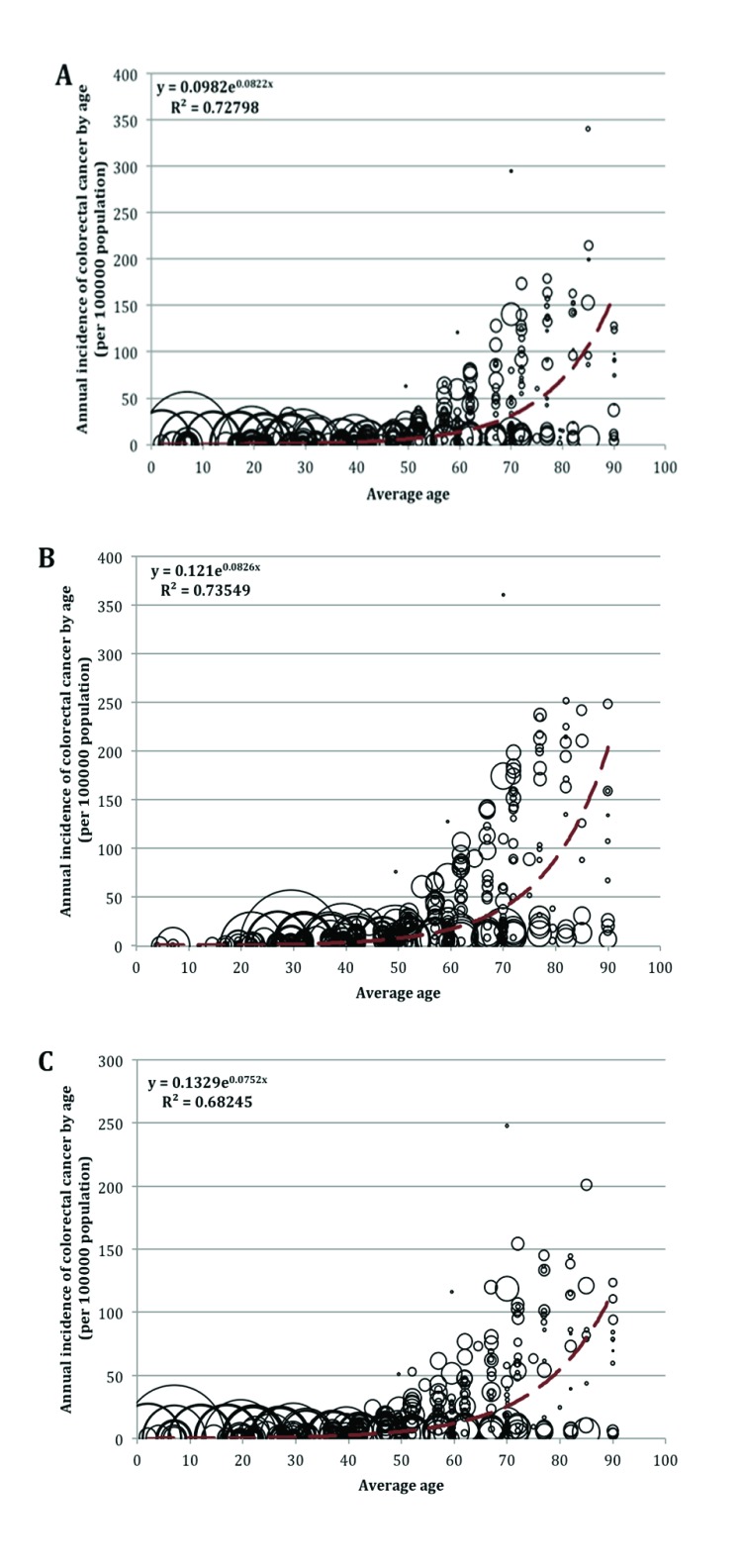
Incidence of colorectal cancer by age groups for both sexes (**A**), men (**B**) and women (**C**).

**Figure 7:**
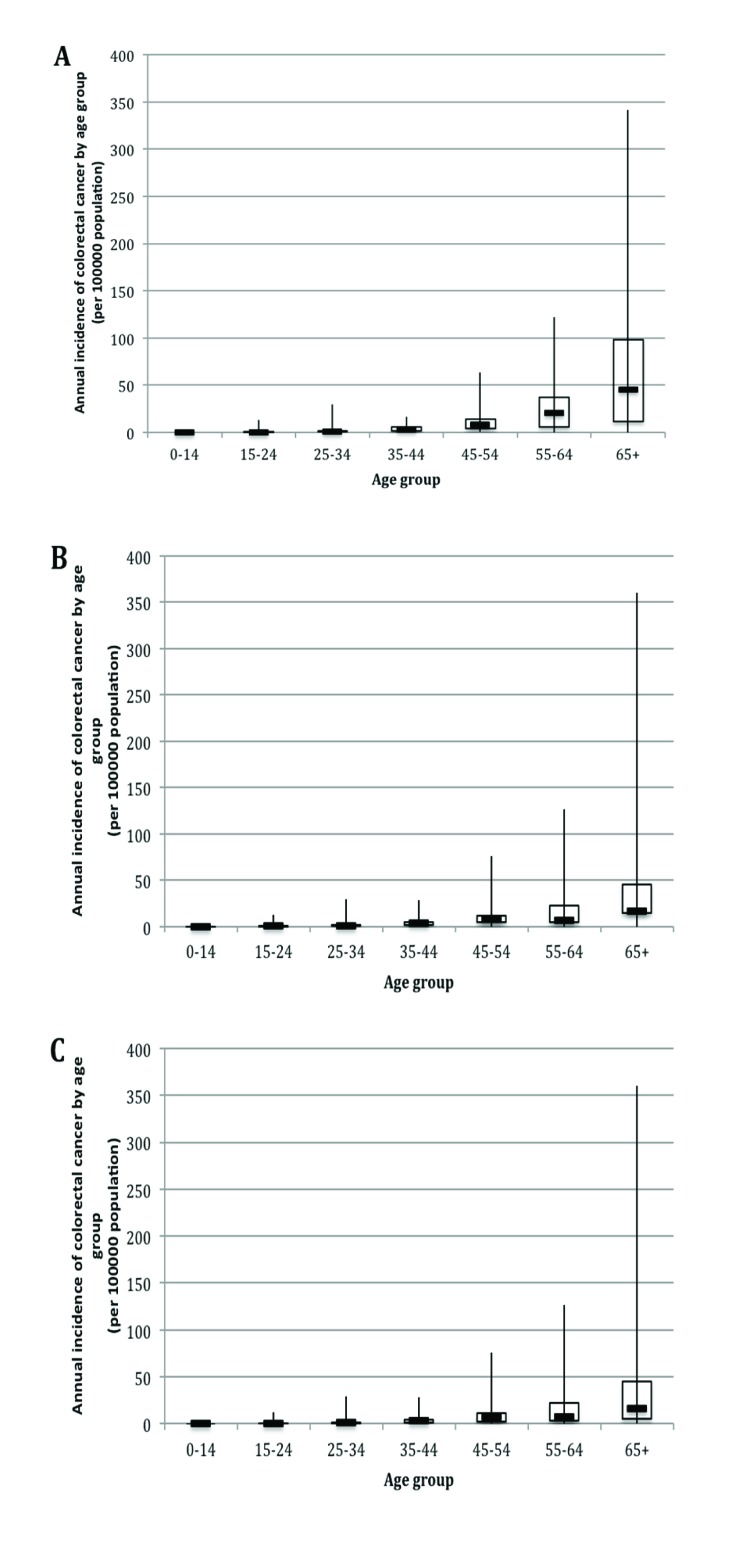
Crude incidence of colorectal cancer by age group for both sexes (**A**), men (**B**) and women (**C**).

**Figure 6** show the distribution of incidence by age, calculated from all 57 data sets, producing 601 data points. The age–related distribution of incidence shows the predicted pattern, based on the biology of CRC, of substantial increase with age. It can also be noted that the calculated incidences are much higher in males than females. There were large variations in reported all–age incidence, owing to CRC being predominantly a disease of the elderly. As such, incidences were separated into age groups that were decided based on the ranges used by the primary data sources. They were selected to ensure that the majority of data points were the median in the ranges chosen. **Figure 7** shows the box–and–whisker plots produced from allocating the individual data points into age groups. The values used for these graphs are presented in Online Supplementary Document(Online Supplementary Document). The median values of incidence for males, females and both sexes by age group are shown in **Table 2**. As expected, the incidence increases with age, with the incidence in people below 35 years being negligible. Again, it is also evident that the incidence of CRC is significantly higher in males than females.

**Table 2:** Crude annual incidence of colorectal cancer in Sub–Saharan Africa (per 100 000 population)

	Age (years)							
	**0–14**	**15–24**	**25–34**	**35–44**	**45–54**	**55–64**	**65–74**	**75+**
Both sexes	0.00	0.26	1.15	3.48	8.72	22.11	34.37	86.87
Men	0.00	0.31	1.22	3.40	8.84	21.13	37.00	103.48
Women	0.00	0.10	0.91	2.91	8.09	15.65	23.48	71.36

**Figure 8:**
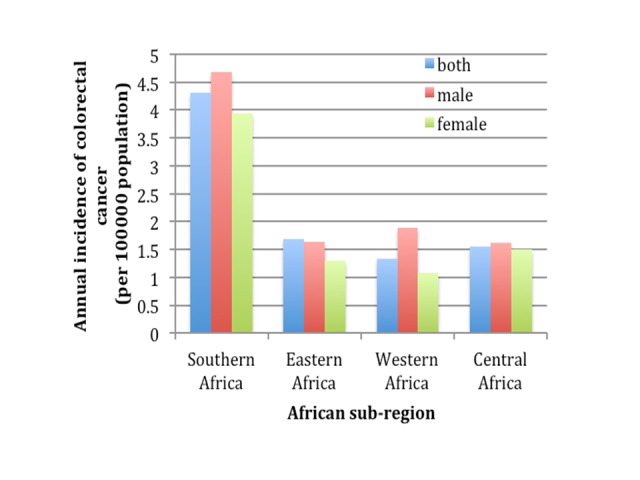
Annual crude incidence of colorectal cancer by African sub–region (per 100 000 population).

**Figure 8** shows the crude incidence of CRC in SSA by African sub–regions. The data used for these figures are presented in Online Supplementary Document(Online Supplementary Document). The crude incidence of CRC in SSA was found to be 4.04 cases per 100 000 population. This incidence was significantly higher in Southern Africa, which is discussed later in the text. The median incidence values calculated in **Table 2** were combined with the UN ESA 2000 estimates for SSA. This produced **Table 3** showing the estimated number of new cases of CRC reported in that year. The reason for using the 2000 population data are considered in the discussion section of this review. It is apparent that the majority of diagnoses of CRC occur in age groups older than 55 years, again with CRC more frequent in males than females. **Table 4** shows a comparison of the estimates produced by this review and those from GLOBOCAN [44]. The estimates are very similar, with the minor difference perhaps explained by the predicted year of the estimates; this will be discussed later in this review.

**Table 3:** Annual number new cases of colorectal cancer in Sub–Saharan Africa in 2000

	Age (years)							
	**0–14**	**15–24**	**25–34**	**35–44**	**45–54**	**55–64**	**65–74**	**75+**
Both sexes	0	456	1428	2791	4651	7808	6732	1099
Men	0	267	762	1371	2294	3514	3321	3069
Women	0	67	565	1158	2216	2922	2492	1575

**Table 4:** Comparison of estimates of annual new cases of colorectal cancer in Sub–Saharan Africa

	Current review’s estimate	G LOBOCAN estimate
Both sexes	23 147	24 711
Men	11 300	13 666
Women	9536	11 045

GLOBOCAN – WHO project to provide contemporary estimates of the incidence of, mortality, prevalence and disability-adjusted life years (DALYs) from major type of cancers, at national level, for 184 countries of the world

### Subsidiary information

**Figure 9:**
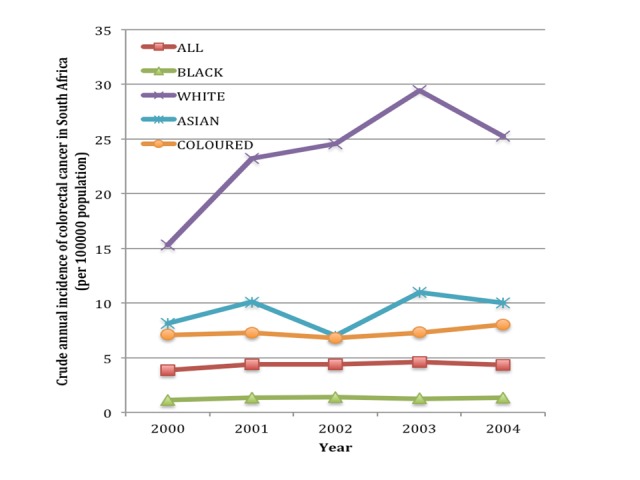
Crude incidence of colorectal cancer in South Africa (per 100 000 population) by ethnicity for 2000–2004.

**Figure 10:**
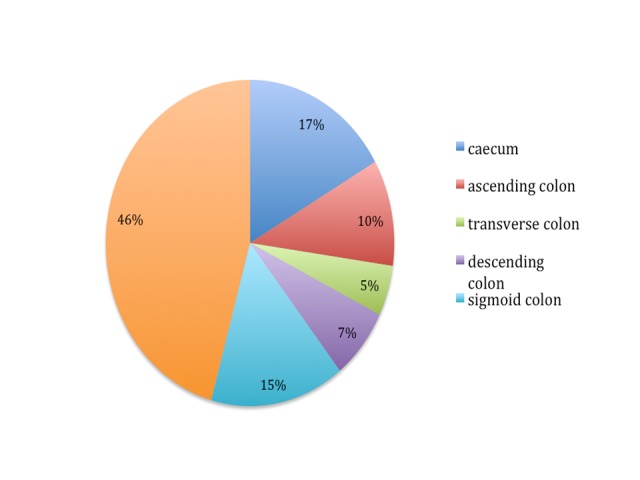
Colorectal cancer by anatomical tumour site.

**Figure 11:**
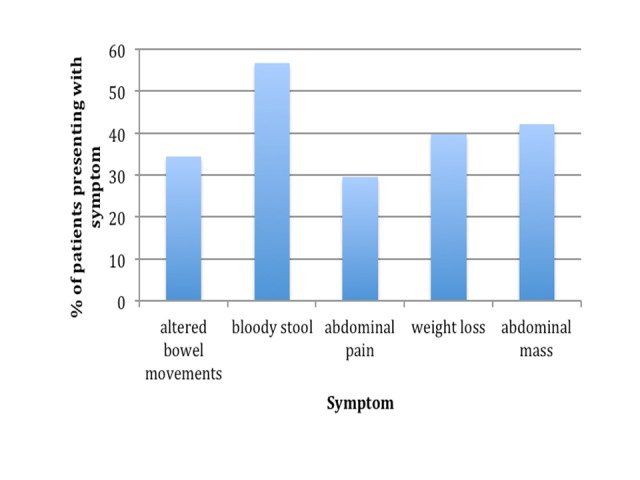
Presenting symptoms in colorectal cancer.

**Figure 12:**
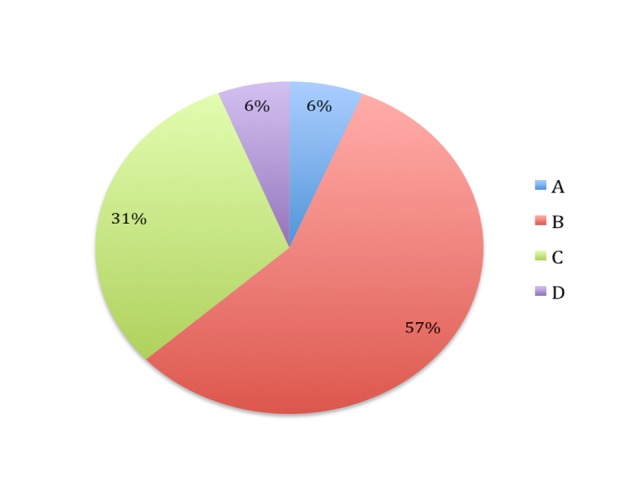
Duke's staging of the colorectal cancer.

Owing to the high standard of data available from the South African Cancer Registry for 2000–2004, a separate analysis was carried out to assess the relationship between incidence of CRC and ethnicity over time (**Figure 9**). Although the data refers to a relatively short time period, the differences in incidence between different ethnic groups can be observed very clearly. Two of the data sets in this review, with further six found through the systematic review, contained information on the nature of the cases of CRC diagnosed in SSA. Five papers contained information on the anatomical sub–site of CRC, and the findings are presented in **Figure 10**. Although the percentage of cases in each anatomical location varied between the papers, all reported the rectum as the most common site, with one paper reporting this to include as high as 60% of cases. Data regarding the symptoms at presentation were obtained from five papers, shown in **Figure 11**. The most common presenting symptom was found to be bloody stool with nearly 57% of patients reporting this. It must be remembered that some patients may have recognised more than one of the symptoms listed. In addition, some patients may not have noticed less dramatic symptoms, such as altered bowel movements, until prompted by a medical professional. **Figure 12** presents information from four papers that contained data on the Duke’s Stage of the case of CRC diagnosed. It can clearly be seen that the majority of cases were diagnosed at Stage B, with very few at the first or last stage.

## DISCUSSION

This study found the incidence of CRC in SSA to be higher in males than females with the peak in the 75+ age group and a crude incidence of 4.04 per 100 000 population. On the basis of the incidence calculated by this review, it was estimated that there were about 23 000 new cases of CRC in 2000, with nearly 59% occurring in males. Based on the available evidence, the incidence of CRC in SSA appears to be much lower than in high–income countries. However, the trends associated with sex and age were very similar. The male:female ratio in the UK is estimated to be 1.26 while this review estimated the SSA ratio to be 1.19 [47]. One of the greatest concerns related to these overall conclusions may be that the incidence of CRC in Africa is systematically under–reported and that it is generally of poorer quality than in high income countries. However, the data from South Africa in the years 2000–2004 provide the best available evidence that the overall conclusions are likely to be true. This is because South Africa has registries of the highest qualities and its diverse demographic structure offers a direct comparison of the rates in different ethnic populations. In South Africa, incidence is highest in whites, followed by the Asian and then coloured populations, with blacks having the lowest reported incidence [27-31].

This review also found that the major anatomical site of CRC was the rectum (in 46% of cases), followed by the caecum (17%). These trends are fairly similar to those found in the Western world. A study looking at anatomical sub–site of CRC in Europe found the most common sites to be rectum (31%), sigmoid colon (21%) and caecum (10%) [48]. Although the presenting symptoms reported by papers in this review were not very different from those elsewhere in the world, the signs associated with the more advanced stages of the disease, such as rectal bleeding, appear to be more common [49]. Moreover, this review found that a majority (57%) of cases of CRC were at Duke’s Stage B at diagnosis, with very few cases being found at the most treatable Stage A. This does not support concerns that in Africa, because of less developed and less efficient health systems, cancer would generally be diagnosed at a later stage – at least it is not true for CRC and in registries that likely over-represent urban areas. However, this may also be due to lack of expert knowledge of the condition, leading to incorrect staging of tumours. This highlights the need for further research into this area in order to improve public health service provision.

In terms of the quality of primary data used in this study, there were two main sources of potential limitation: incomplete data and systematic bias. Although the problem of incomplete data was only encountered in 9 of the 57 data sets, it should not have affected the overall conclusions. Twenty–one of those data sets were obtained from direct contact with cancer registries in SSA. This review should not be affected by a major language bias, as data sets have been retrieved in all major languages used in Africa, including French and Dutch. It is also unlikely that this review is limited by the conventional form of publication bias, as unpublished registry data was also included. It is important to note that the latter may provide more recent data than that from published papers owing to the usual delay in the release of articles. The majority of data sets eligible for inclusion in this review contained data from the late 1990s and early 2000s. As such, any estimates produced by this review essentially refer to a mid–point of the year 2000, and are based on the correct population denominator from UN ESA. This was the best estimate that could be produced with the data available However, it is recognised that rates of NCDs are increasing in the developing world and therefore the estimates for 2000 are likely to be lower than the true current level.

This review made a comparison between the estimated number of new cases of CRC in Africa and estimates produced by GLOBOCAN [44]. Although it appears that this review underestimates the incidence in comparison to GLOBOCAN, it should be noted that there is an 8–year difference, with GLOBOCAN reporting the estimates for the year 2008. However, a short analysis of the data sources used by GLOBOCAN found that the majority of their data points also came from the 1990s. This probably explains the great similarity between the two estimates, which are very supportive of each other. However, because this review uses the same primary data with an additional 36 data sets it could be viewed as more comprehensive.

Finally, this review used information from 57 mutually exclusive cancer registry data sets, 15 of which came from articles published in peer–reviewed journals and 42 from unpublished data sources. The assumption is that the peer–reviewed articles would be of a higher quality so it was important to explore differentiating incidence across these two sources in a simple sensitivity analysis. The results are presented in **Table 5**. Although there are modest differences in incidence, this is partly driven by the inclusion of the South African data sets. The difference in incidence between published and unpublished data sets was significantly lower with the exclusion of South African data from both.

**Table 5:** Sensitivity analysis of published vs unpublished data sets

	Crude incidence of CRC/100 000 population	
	**Published data sets**	**Unpublished data sets**
With South Africa	1.11	4.28
Without South Africa	1.07	2.05

In terms of the validity and reliability of case definitions used to measure the frequency of CRC in the population in Africa, the current National Institute for Clinical Excellence (NICE) provides clinical guidelines that recommend the use of colonoscopy, biopsy, sigmoidoscopy, CT scan and barium enema in the diagnosis of CRC [50]. However, in many resource–poor settings, such as those found in a number of SSA countries, such extensive diagnostic tests are not feasible. Many registries only document histologically–diagnosed cancers, but even this is lacking in some areas [51]. This may result in the number of cases of CRC being underestimated in some settings. Another possible point of concern comes from the notion that some data sets included in this review did not specify whether ICD or another classification system was used as their case definition. Earlier versions of the ICD that preceded ICD–10 were recognized to be less specific [52]. This concern should be noted in regard to interpreting any fluctuations in the reports from cancer registries that were active over a long period of time. It is estimated that only 1% of the population of Africa are covered by cancer registries [53]. With limited resources and critically low numbers of health workers, the implementation of a cancer registry has not been seen as a priority in tackling the burden of disease in many countries. However, accurate measurement of diseases is vital in achieving effective and efficient intervention programs [54].

Although data for 19 different countries in SSA was obtained, over 75% of the data points came from countries in Southern and Eastern Africa. These regions were found to have the highest incidences of CRC. Given that the majority of these data sets came from South Africa and Zimbabwe, it is important to recognise the differences in the population of these countries in comparison to the rest of SSA. These two countries had the highest incidence of CRC of the countries studied. South African law previously segregated its people into 4 ethnic categories; “Black”, “Coloured” (mixed–race), “White” and “Asian/Indian”. Although this law was abolished over 10 years ago, much of the population still identify themselves based on these groups and many epidemiology studies still separate the population in this way [55]. This can still be seen as evident in the data provided by the South African Cancer Registry for 2000–2004. Similarly, the data sets from Zimbabwe also separate their population into “African” and “European”. This separation between ethnic groups allows for comparison between the groups, especially given the well–documented variation in incidence of CRC between white and black populations, which is consistently shown to be substantial.

South Africa is also known to have the highest numbers of people infected with HIV/AIDS of any country in Sub–Saharan Africa [56]. There is evidence to suggest an increased risk of developing CRC amongst HIV/AIDS infected populations, however the biological mechanism behind this is still poorly understood [57]. Karposi’s sarcoma is strongly associated with HIV/AIDS infection and there are documented cases of involvement of the colorectum [58,59]. It is also possible that this may account for the higher incidence of CRC in South Africa. However, countries like Swaziland and Botswana, with higher percentages of HIV/AIDS infected populations had much lower incidence of CRC.

Ideally, cancer registries should be population–based, however, in developing countries, this is often not possible. Problems such as the limited health system infrastructure and cultural and religious obstacles in the reporting of diseases like cancer have contributed to the lack of routine data collection [54]. The alternative option of data collection through a hospital–based registry is better than no registry at all. This type of data will only cover people who have presented and been recognised as symptomatic at a local health facility, have been referred to centres where there are cancer services, and who can access these health care facilities, both physically and financially. However, it can still provide valuable information on cancer, particularly if adjustments can be made for selection biases in the population using the hospital. Because of this triaging system from primary to secondary care, those seeking medical attention are likely to be from higher socioeconomic backgrounds with higher levels of education and a greater awareness of the risk factors for developing diseases such as cancer. However, these people are also more likely to live a ‘Westernised’ lifestyle; increasing their risk of developing CRC. Hospital–based studies are also often based in urban areas where the risk profile is very different to those people living in rural areas. This highlights the need for national, population–based studies or more sophisticated data adjustments to ensure that any estimates produced are an accurate representation of the whole country.

**Figure 13:**
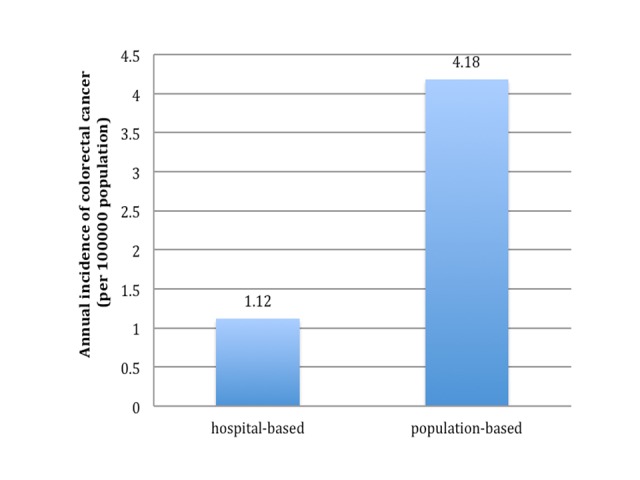
Comparison of incidence of colorectal cancer (per 100 000 population) in Sub–Saharan Africa between hospital–based and population–based cancer registries.

To illustrate the difference between hospital–based and population–based cancer registries, a comparison of the crude incidence of CRC (per 100 000 population) is presented in **Figure 13**. Again, ideally a more sophisticated subgroup analysis would be undertaken to account for the influence of this apparent difference. Adjustments should be made allowing for the differences in the source of the data (published/unpublished), geographical coverage (particularly with reference to the dominance of South Africa) and the ethnic group of patients.

In many urban areas of SSA, there has been an increase in health risk behaviours associated with CRC, such as smoking, decreased physical activity, alcohol consumption and poor diet. There is also evidence of the increase in the health consequences of these behaviours, in terms of increased rates of obesity, diabetes and hypertension [51]. It is important that health education programs are set up to enable people to be more aware of the consequences of these unhealthy behaviours. Prevention is a more cost–effective and long–term solution to dealing with the burden of cancer than treating the cases once diagnosed [60,61]. Although screening for CRC is thought to be cost–effective in high–income countries, whether this is the case in LMICs is debated. Implementing a screening program for CRC requires the purchase and maintenance of expensive equipment, skilled specialists and education of the public to maximise utilisation. Lambert et al. argue that this is not feasible in resource–poor settings like SSA [62]. However, with more accurate data from population–based studies it may be that the burden of disease attributable to CRC is greater than first thought. In this case, screening programmes, particularly if they are linked to a more generic family health programme engaging both men and women, may be a more cost-effective option to treating more advanced cancers.

In low–resource countries the national budget devoted to health systems is extremely low and as a consequence the public health service provision can be very weak [63]. This has led to the treatment options for patients with all forms of cancer including CRC in SSA being severely limited in comparison to patients in the Western world. For instance, a surgical procedure commonly performed in high–income world as a treatment, such as resection, was noted to only be attempted in very few countries that have specialist cancer hospitals and only in cases where the patients themselves were able to afford the cost of the procedure [24]. Cultural factors are also relevant to the acceptance of treatment, with some patients refusing to undergo surgery because of fear that a colostomy could result in rejection from their community [24,49]. It is estimated that only 18% of the need for radiation treatment in cancer patients is met in Africa as a whole, and this is likely to be even lower in the Sub–Saharan region [64]. Delays in seeking medical attention mean that for many patients with advanced stage of cancers palliative treatment was the only available option [49]. Follow–up of patients in all clinical settings is notoriously poor. One study reported that less that 30% of patients who received adjuvant treatment were seen again after six months. Although the reasons for this will be multifactorial, it was suggested that the treatment for many of these patients was unsuccessful and they had died [49].

In order to improve the volume and quality of information available on cancer in SSA there needs to be stronger investment in cancer registries. Being able to counter the arguments that it is not effective, efficient or ethical to invest scarce resources in setting–up cancer registries purely for epidemiological research purposes is important as such data sources will become an invaluable source of evidence and guidance for policy setting, programme implementation and improving practice.

In summary, this systematic analysis has highlighted the lack of data on CRC, mirroring the lack of data for all cancers in SSA. There is a notable lack of any recent published data. Greater use could be made of cancer registry data through direct contact and open access to their databases. All NCDs, including CRC, are increasing in low–income regions such as SSA. It is vital that the burden of disease attributable to this is accurately and regularly monitored. More information on the dynamics of this burden is also required, including who is affected, where and whether they have adequate access to treatment.
