# Supplementary material for: Estimating the incidence of colorectal cancer in Sub–Saharan Africa: A systematic analysis
Source: J Glob Health. 2012 Dec;2(2):020404. doi: 10.7189/jogh.02.020204 (PMC3529315; doi:10.7189/jogh.02.020204)
Supplement: Online Supplementary Document [file jogh-02-020404-s001.pdf]

Online Supplementary Document

Graham et al. Estimating the incidence of colorectal cancer in sub-Saharan Africa: A systematic analysis
Journal of Global Health 2012;2:020404

1. **Data from the Systematic Review**
2. **Data from IARC**
3. **Data from Botswana Cancer Registry**
4. **Data from South African Cancer Registry**
5. **Crude Incidence of CRC by Paper (Both Sexes)**
6. **Crude Incidence of CRC by Paper (Males Only)**
7. **Crude Incidence of CRC by Paper (Females Only)**
8. **Data for Graphs**
9. **Crude Incidence of CRC by SSA Country**

Data from Systematic Review (extract)				
	**Average age**	**Population size of age group**	**Cases in age group**	**Incidence in age group (/100000/year)**
**1. Rwanda**				
**MALE**	7	201597.5061	0	0
	19.5	77088.33259	1	0.518885267
	29.5	49817.68976	0	0
	39.5	35092.40613	2	2.279695491
	49.5	22431.06311	0	0
	59.5	13673.30574	1	2.925408146
	70	8371.445449	3	14.33444209
**FEMALE**	7	192881.11	0	0
	19.5	79087.04722	0	0
	29.5	52101.14357	0	0
	39.5	35646.47348	2	2.244261274
	49.5	22205.66634	1	1.801342026
	59.5	14129.99905	1	2.830856525
	70	12020.30922	3	9.983104246
**BOTH**	7	394235.7582	0	0
	19.5	156231.0684	1	0.256031021
	29.5	101982.4554	0	0
	39.5	70754.31714	4	2.261346112
	49.5	44630.4494	1	0.896249098
	59.5	27816.02926	2	2.87603954
	70	20493.42	6	11.71107604
Full dataset is available from the corresponding author.				

Data from IARC (extract)				
	**Average age**	**Population size of age group**	**Cases in age group**	**Incidence in age group (/100000/year)**
**16. Mali**				
**MALES**	7	153211	0	0
	19.5	74059	3	0.810164869
	29.5	59718	6	2.009444389
	39.5	36556	14	7.659481344
	49.5	21415	6	5.603548914
	59.5	10737	10	18.62717705
	70	7662	9	23.49256069
**FEMALES**	7	163114	0	0
	19.5	75709	2	0.528338771
	29.5	43852	5	2.280397701
	39.5	27171	2	1.472157815
	49.5	16504	5	6.059137179
	59.5	9206	3	6.517488594
	70	7711	4	10.37478926
**BOTH**	7	316325	0	0
	19.5	149768	5	0.667699375
	29.5	103570	11	2.12416723
	39.5	63727	16	5.021419493
	49.5	37919	11	5.801840766
	59.5	19943	13	13.03715589
	70	15373	13	16.91276914
Full dataset is available from the corresponding author.				

Data from Botswana Cancer Registry (extract)				
	**Average age**	**Population size of age group**	**Cases in age group**	**Incidence in age group (/100000/year)**
**37. Botswana**				
**MALE**	4	330000	0.463815789	0.010039303
	17	112000	4.174342105	0.266220798
	22	107000	2.463815789	0.164473684
	27	90000	6.957236842	0.552161654
	32	68000	8.8125	0.925682773
	37	53000	14.37828947	1.937774862
	42	43000	12.05921053	2.003191117
	47	38000	12.05921053	2.266768896
	52	33000	15.30592105	3.312969925
	57	23000	17.625	5.473602484
	62	17000	10.66776316	4.482253428
	67	12000	14.37828947	8.558505639
	72	8000	8.8125	7.868303571
	80	3000	7.884868421	18.77349624
**FEMALE**	4	325011	0.536184211	0.01178387
	17	110599	4.825657895	0.311657293
	22	104330	8.578947368	0.587349712
	27	82813	8.042763158	0.693711232
	32	60209	10.1875	1.208587705
	37	48393	16.62171053	2.453381765
	42	43426	13.94078947	2.293028777
	47	40916	13.94078947	2.433695075
	52	36114	17.69407895	3.499647732
	57	24558	20.375	5.926203856
	62	20182	12.33223684	4.364651968
	67	16094	16.62171053	7.377066221
	72	11283	10.1875	6.449335916
	80	5123	17.53618421	24.45021641
Full dataset is available from the corresponding author.				

Data from South African Cancer Registry (extract)				
	**Average age**	**Population size of age group**	**Cases in age group**	**Incidence in age group (/100000/year)**
**38. South Africa**				
**MALES**	2	53662	0	0
	7	47571	0	0
	12	51648	0	0
	17	55085	0	0
	22	50505	0	0
	27	51062	2	3.916807019
	32	47603	0	0
	37	44899	2	4.454442192
	42	40237	3	7.455824241
	47	35011	1	2.85624518
	52	28588	8	27.98376941
	57	21258	10	47.04111393
	62	15440	14	90.67357513
	67	9366	6	64.06149904
	72	5373	7	130.2810348
	77	2899	3	103.48396
	82	1398	3	214.5922747
	90	714	0	0
**FEMALES**	2	52425	0	0
	7	45928	0	0
	12	49921	0	0
	17	53850	0	0
	22	50309	0	0
	27	51590	0	0
	32	48309	1	2.070007659
	37	45992	3	6.522873543
	42	41812	1	2.391657897
	47	37076	3	8.091487755
	52	31080	1	3.217503218
	57	24059	7	29.09514111
	62	18476	4	21.64970773
	67	12236	4	32.69042171
	72	7679	4	52.0901159
	77	4395	6	136.5187713
	82	2198	0	0
	90	1218	0	0
**BOTH**	2	106087	0	0

Data from South African Cancer Registry (extract)				
	**Average age**	**Population size of age group**	**Cases in age group**	**Incidence in age group (/100000/year)**
	7	93499	0	0
	12	101569	0	0
	17	108935	0	0
	22	100814	0	0
	27	102652	2	1.948330281
	32	95912	1	1.042622404
	37	90891	5	5.501094718
	42	82049	4	4.87513559
	47	72087	4	5.548850694
	52	59668	9	15.08346182
	57	45317	17	37.5135159
	62	33916	18	53.07229626
	67	21602	10	46.29201
	72	13052	11	84.27827153
	77	7294	9	123.3890869
	82	3596	3	83.42602892
	90	1932	0	0
Full dataset is available from the corresponding author.				

Crude Incidence of CRC by Paper (Both Sexes)				
		**BOTH SEXES**		
**Study No.**	**Country**	**total population**	**average annual no. cases**	**incidence of CRC (/100000) all ages**
1	Rwanda	816143.4978	3.5	0.428846154
2	Zimbabwe	1253030	12.375	0.987606043
3	Gambia	867387	6.1	0.703261635
4	Ivory Coast	2852804	20	0.701064637
5	Malawi	742952	5	0.672990987
6	South Africa	1079250	8.2	0.759786889
7	Guinea	994312	11	1.106292592
8	South Africa	2007407.407	25.2	1.255350554
9	Uganda	1034397	25.437661	2.459177763
10	Mali	646141	10.5	1.625032307
11	Rwanda	4353600	5.333333333	0.122503981
12	Ghana	2889330.934	29.63	1.025496929
13	South Africa	381756.76	14.12	3.698690234
14	Gambia	774000	12.5775	1.625
15	Uganda	1073500	53.33333333	4.968172644
16	Mali	706625	13.8	1.952945339
17	Mali	793652	17.4	2.192396668
18	Niger	515662	8.714285714	1.689922025
19	Nigeria	1497843	26.5	1.769210792
20	Congo	603614	14.75	2.443614628
21	Kenya	617537	11.33333333	1.835247659
22	Malawi	882190	12	1.360251193
23	Uganda	1140957	25.2	2.208672194
24	Zimbabwe	1128129	32.5	2.880876212
25	Zimbabwe	1357731	33.25	2.448938707
26	Zimbabwe	40188	31	77.13745397
27	Namibia	1596407	38	2.380345363
28	South Africa	260821	5	1.917023553
29	South Africa	541547	4	0.738624718
30	South Africa	28313752	431.5	1.523994418
31	South Africa	5061252	1141.5	22.55370805
32	South Africa	3276003	131	3.998775337
33	South Africa	983503	69.75	7.091996669
34	Swaziland	926820	14.75	1.591463283
35	Cameroon	1078900	11.5	1.065900454
36	Tanzania	840386	7.666666667	0.912279199
37	Botswana	1866051	21.71428571	1.163649103
38	South Africa	1140872	93	8.151659432
39	South Africa	35070765	401	1.143402489

Crude Incidence of CRC by Paper (Both Sexes)				
		**BOTH SEXES**		
**Study No.**	**Country**	**total population**	**average annual no. cases**	**incidence of CRC (/100000) all ages**
40	South Africa	3989852	284	7.118058514
41	South Africa	6797843	1041	15.31368112
42	South Africa	1129685	114	10.09130864
43	South Africa	34636147	479	1.382948282
44	South Africa	4036856	295	7.307667155
45	South Africa	4634660	1078	23.25952713
46	South Africa	1159247	81	6.987294338
47	South Africa	36236232	522	1.44054713
48	South Africa	4086363	279	6.82758727
49	South Africa	4714020	1159	24.58623425
50	South Africa	1140097	125	10.96397938
51	South Africa	36914284	477	1.292182723
52	South Africa	4131096	303	7.334615318
53	South Africa	4244346	1250	29.45094486
54	South Africa	1137986	114	10.01769793
55	South Africa	38468136	526	1.367365448
56	South Africa	4150970	335	8.070402822
57	South Africa	4496847	1136	25.26214479
**TOTAL**		**308111885.6**	**12437.1354**	**4.036564631**

Crude Incidence of CRC by Paper (Males Only)				
		MALES		
Study No.	Country	total population	Average annual no. cases	incidence of CRC (/100000) all ages
1	Rwanda	408071.7489	2.8	0.686153846
2	Zimbabwe	650600	20	3.07408546
3	Gambia	427179	0.9	0.210684514
4	Ivory Coast	1403786	12.33333333	0.878576459
5	Malawi	379374	3	0.790776384
6	South Africa	392750	5.2	1.323997454
7	Guinea	507469	6	1.182338231
8	South Africa	1000000	11.6	1.16
9	Uganda	506430	7.6407	1.508737634
10	Mali	323363	7.5	2.31937482
11	Rwanda	2357200	2.666666667	0.113128571
12	Ghana	1314350.079	16.44	1.250808309
13	South Africa	190878.38	5.34	2.797592897
14	Gambia	385000	8.983333333	2.333333333
15	Uganda	526900	12.85714286	2.440148578
16	Mali	363358	9.6	2.642022468
17	Mali	412874	9.8	2.373605507
18	Niger	260495	5.285714286	2.029103931
19	Nigeria	743186	14.5	1.951059358
20	Congo	302308	8.5	2.811701973
21	Kenya	313020	8	2.555747237
22	Malawi	449314	5.5	1.224088277
23	Uganda	562179	13.2	2.348006596
24	Zimbabwe	593784	21	3.536639586
25	Zimbabwe	697945	20.25	2.90137475
26	Zimbabwe	19102	16.25	85.06962622
27	Namibia	794765	21.25	2.673746328
28	South Africa	119325	3.333333333	2.793491166
29	South Africa	241605	2	0.827797438
30	South Africa	14211421	227.25	1.599065991
31	South Africa	2516346	586.25	23.29767051
32	South	1601090	62.75	3.919205042

Crude Incidence of CRC by Paper (Males Only)				
		MALES		
Study No.	Country	total population	Average annual no. cases	incidence of CRC (/100000) all ages
	Africa			
33	South Africa	487385	38	7.796711019
34	Swaziland	438822	9	2.050945486
35	Cameroon	561600	5.5	0.979344729
36	Tanzania	420193	3.666666667	0.872614886
37	Botswana	937000	12.36752392	1.319906502
38	South Africa	562319	59	10.49226507
39	South Africa	16946863	230	1.357183332
40	South Africa	1943285	151	7.770347633
41	South Africa	2283802	572	25.04595407
42	South Africa	546670	54	9.877988549
43	South Africa	17736334	250	1.409535928
44	South Africa	1965315	166	8.446483134
45	South Africa	2264195	568	25.08617853
46	South Africa	571044	33	5.778889192
47	South Africa	17506462	282	1.61083376
48	South Africa	1988617	143	7.190927162
49	South Africa	2356705	619	26.26548507
50	South Africa	556278	72	12.9431687
51	South Africa	17562179	251	1.429207617
52	South Africa	1979934	156	7.879050514
53	South Africa	2051917	625	30.4593217
54	South Africa	557485	68	12.1976376
55	South Africa	19031558	279	1.465986127

Crude Incidence of CRC by Paper (Males Only)				
		**MALES**		
**Study No.**	**Country**	**total population**	**Average annual no. cases**	**incidence of CRC (/100000) all ages**
56	South Africa	2036176	203	9.969668634
57	South Africa	2206802	578	26.19174715
**TOTAL**		**150474408.2**	**6585.514414**	**4.376501289**

Crude Incidence of CRC by Paper (Females Only)				
		**FEMALE**		
**Study No.**	**Country**	**total population**	**Average annual no. cases**	**incidence of CRC (/100000) all ages**
1	Rwanda	408071.7489	2.8	0.686153846
2	Zimbabwe	602430	13	2.157927062
3	Gambia	440208	3	0.68149602
4	Ivory Coast	1449018	7.666666667	0.529093957
5	Malawi	363578	2	0.550088289
6	South Africa	686500	3	0.436999272
7	Guinea	486843	5	1.02702514
8	South Africa	1007407.407	13.6	1.35
9	Uganda	527967	4.660343	0.882695888
10	Mali	322778	3	0.929431374
11	Rwanda	1996400	4	0.200360649
12	Ghana	1336580.835	13.19	0.986846411
13	South Africa	190878.38	5.956	3.120311478
14	Gambia	389000	7.78	2
15	Uganda	546600	10	1.829491401
16	Mali	343267	4.2	1.223537363
17	Mali	380778	7.6	1.995913629
18	Niger	255167	3.428571429	1.343657851
19	Nigeria	754657	12	1.590126375
20	Congo	301306	6.25	2.0743032
21	Kenya	304517	3.333333333	1.094629638
22	Malawi	432876	6.5	1.501584749
23	Uganda	578778	12	2.073333817
24	Zimbabwe	534345	11.5	2.152167607
25	Zimbabwe	659786	13	1.970335836
26	Zimbabwe	21086	14.75	69.95162667
27	Namibia	801642	16.75	2.089461381
28	South Africa	141496	1.666666667	1.177889599
29	South Africa	299942	2	0.66679558
30	South Africa	14102331	204.25	1.448342122
31	South Africa	2544906	555.25	21.81809466
32	South	1674913	38	2.268774557

Crude Incidence of CRC by Paper (Females Only)				
		**FEMALE**		
**Study No.**	**Country**	**total population**	**Average annual no. cases**	**incidence of CRC (/100000) all ages**
	Africa			
33	South Africa	496118	31.75	6.399687171
34	Swaziland	487998	5.75	1.178283518
35	Cameroon	517300	6	1.159868548
36	Tanzania	420193	4	0.951943512
37	Botswana	929051	15.58373206	1.677381765
38	South Africa	578553	34	5.876730395
39	South Africa	18123902	171	0.943505433
40	South Africa	2046567	133	6.498687803
41	South Africa	4514041	469	10.38980373
42	South Africa	583015	60	10.29133041
43	South Africa	16899813	229	1.355044579
44	South Africa	2071541	129	6.227248218
45	South Africa	2370465	510	21.51476609
46	South Africa	588203	48	8.160448009
47	South Africa	18729861	240	1.281376301
48	South Africa	2097746	136	6.483149056
49	South Africa	2357315	540	22.90741797
50	South Africa	583819	53	9.078156072
51	South Africa	19352105	226	1.167831613
52	South Africa	2151162	147	6.833516025
53	South Africa	2192429	625	28.50719453
54	South Africa	580501	46	7.924189622
55	South Africa	19436578	247	1.270799829

Crude Incidence of CRC by Paper (Females Only)				
		**FEMALE**		
**Study No.**	**Country**	**total population**	**Average annual no. cases**	**incidence of CRC (/100000) all ages**
56	South Africa	2114794	132	6.241742695
57	South Africa	2290045	558	24.36633341
**TOTAL**		**157399168.4**	**5807.215313**	**3.689482844**

Data For Graphs							
	**Both Sexes**						
	**0-14**	**15-24**	**25-34**	**35-44**	**45-54**	**55-64**	**65+**
**lower quartile**	0.00	0.16	0.74	1.95	4.06	4.09	10.55
**minimum**	0.00	0.00	0.00	0.26	0.68	0.00	0.00
**median**	0.00	0.37	1.23	3.55	7.40	7.20	16.39
**maximum**	0.27	13.00	29.59	16.19	63.07	121.59	295.23
**upper quartile**	0.04	0.62	1.78	4.97	11.62	22.16	45.33
	**Male Only**						
	**0-14**	**15-24**	**25-34**	**35-44**	**45-54**	**55-64**	**65+**
**lower quartile**	0.00	0.03	0.68	2.00	4.51	4.33	14.20
**minimum**	0.00	0.00	0.00	0.00	0.00	0.00	0.00
**median**	0.00	0.39	1.33	3.25	7.66	9.30	21.13
**maximum**	0.35	7.00	4.66	28.64	76.22	127.15	360.62
**upper quartile**	0.00	0.79	2.06	5.22	11.99	21.13	47.39
	**Female Only**						
	**0-14**	**15-24**	**25-34**	**35-44**	**45-54**	**55-64**	**65+**
**lower quartile**	0.00	0.00	0.19	1.62	2.43	3.23	5.72
**minimum**	0.00	0.00	0.00	0.00	0.00	0.00	0.00
**median**	0.00	0.23	0.90	3.21	6.16	8.72	10.71
**maximum**	0.18	6.00	12.40	8.93	51.28	116.30	247.91
**upper quartile**	0.00	0.60	1.50	4.50	12.85	21.54	28.63

Crude Incidence of CRC by SSA Country				
		**incidence of CRC all ages (/100000 population)**		
		**both**	**male**	**female**
Southern Africa	Botswana	1.16	1.32	1.68
	Namibia	2.38	2.67	2.09
	South Africa	4.35	4.72	3.97
	Swaziland	1.59	2.05	1.18
Eastern Africa	Kenya	1.84	2.56	1.09
	Malawi	1.05	1.03	1.07
	Rwanda	0.17	0.20	0.28
	Tanzania	0.91	0.87	0.95
	Uganda	2.33	2.11	1.61
	Zimbabwe	2.89	3.95	2.87
West Africa	Gambia	1.14	1.22	1.30
	Ghana	1.03	1.25	0.99
	Guinea	1.11	1.18	1.03
	Ivory Coast	0.70	0.88	0.53
	Mali	1.94	2.45	1.41
	Niger	1.69	2.03	1.34
	Nigeria	1.77	1.95	1.59
Central Africa	Cameroon	1.07	0.98	1.16
	Congo	2.44	2.81	2.07
